# The Olera.care Digital Caregiving Assistance Platform for Dementia Caregivers: Preliminary Evaluation Study

**DOI:** 10.2196/55132

**Published:** 2024-04-17

**Authors:** Qiping Fan, Minh-Nguyet Hoang, Logan DuBose, Marcia G Ory, Jeswin Vennatt, Diana Salha, Shinduk Lee, Tokunbo Falohun

**Affiliations:** 1 Department of Public Health Sciences Clemson University Clemson, SC United States; 2 School of Public Health Texas A&M University College Station, TX United States; 3 School of Medicine Texas A&M University College Station, TX United States; 4 Internal Medicine George Washington University DC, WA United States; 5 College of Nursing University of Utah Salt Lake City, UT United States; 6 Department of Biomedical Engineering Texas A&M Univesity College Station, TX United States

**Keywords:** evaluation, usability, family caregiver, Alzheimer disease, dementia, digital health, mobile phone

## Abstract

**Background:**

The increasing prevalence of Alzheimer disease and Alzheimer disease–related dementia in the United States has amplified the health care burden and caregiving challenges, especially for caregivers of people living with dementia. A web-based care planning tool, Olera.care, was developed to aid caregivers in managing common challenges associated with dementia care.

**Objective:**

This study aims to preliminarily evaluate the quality and usability of the Olera.care platform and assess the preferences of using the technology and interests in learning about different older adult care services among caregivers.

**Methods:**

For interview 1, we aim to understand caregiving needs and let the participants start engaging with the platform. After they engage with the platform, we schedule the second interview and let the participants complete the Mobile Application Rating Scale. The survey also included sociodemographic characteristics, caregiving experiences, communication preferences in technology adoption, and older adult care service use and interests. Descriptive statistics were used to describe the quality and usability of the platform and characteristics of the participants. We conducted 2-sample 2-tailed *t* tests to examine the differences in the Mobile Application Rating Scale evaluation scores by caregiver characteristics.

**Results:**

Overall, 30 adult caregivers in Texas completed the evaluation. The majority were aged ≥50 years (25/30, 83%), women (23/30, 77%), White (25/30, 83%), and financially stable (20/30, 67%). The Olera.care platform evaluation showed high satisfaction, with an overall mean rating of 4.57 (SD 0.57) of 5, and scored well in engagement (mean 4.10, SD 0.61), functionality (mean 4.46, SD 0.44), aesthetics (mean 4.58, SD 0.53), and information quality (mean 4.76, SD 0.44) consistently across all participants. A statistically significant difference (*P*=.02) was observed in functionality evaluation scores by duration of caregiving, with caregivers dedicating more hours to care rating it higher than those providing less care (mean 4.6, SD 0.4 vs mean 4.2, SD 0.5). In addition, caregivers with less caregiving experience reported significantly higher evaluation scores for aesthetics (*P*=.04) and information quality (*P*=.03) compared to those with longer years of caregiving. All participants expressed a willingness to recommend the app to others, and 90% (27/30) rated the app overall positively. Most of the participants (21/30, 70%) favored anonymous interactions before receiving personalized feedback and preferred computer browsers over mobile apps. Medical home health services were the most used, with a diverse range of services being used. Caregiver support groups, medical providers, memory care, meal services, and adult day care were among the most desired services for future exploration.

**Conclusions:**

The Olera.care web-based platform is a practical, engaging, easy-to-use, visually appealing, and informative tool for dementia caregivers. Future development and research are essential to enhance the platform and comprehensively evaluate it among a broader population.

## Introduction

### Background

With the population of Americans living with Alzheimer disease (AD) and AD-related dementia projected to grow from 6.7 million to 13.8 million by 2060, there is a need for effective and innovative solutions to address the increasing health care burden, particularly with caregivers of people living with dementia [1,2]. Oftentimes, these caregivers are unpaid or informal family members or friends who provide care mostly related to activities of daily living [3,4]. Due to the variability in disease progression and the caregiver’s personal needs, family caregivers encounter diverse unmet needs, including challenges related to the physical and emotional deterioration of the people living with dementia [5-7]. Particularly, caregivers of people living with dementia report difficulties related to assisting the care recipient with activities of daily living, identifying the right older adult living services, navigating the financial and legal aspects of caregiving for people living with dementia, and finding relevant and concise information on dementia and dementia caregiving [5-7]. Due to the multifaceted and individualized burdens that caregivers of people living with dementia face, they are particularly susceptible to experiencing emotional, physical, and financial challenges that increase with disease progression [3,6,7].

To aid informal caregivers with challenges related to dementia care, many digital technologies have been developed that focus on either the needs of the caregiver or of the people living with dementia [8]. These solutions range from web-based training to web-based forums and caregiving groups, psychological and educational forums, and videoconferencing technologies [9,10]. Despite the many digital interventions to aid caregivers and their care recipients, these solutions do not adequately address the individual needs of caregivers of people living with dementia, leading to limited adoption of technologies outside of pilot studies [5,11]. Previous studies have shown that, despite the availability of digital interventions, the unique and individual needs of caregivers of people living with dementia are not always adequately addressed, leading to limited technology adoption and potentially contributing to sustained or increased emotional, physical, and financial burdens associated with caregiving [6,9]. To increase the use of technology that aids caregivers of people living with dementia, technology development should involve caregivers’ feedback on usability and align with caregivers’ expectations and needs [7,12,13]. However, not many studies have investigated the usability and usefulness of technology interventions for dementia care or involved caregivers of people living with dementia in the development process to accurately address caregivers’ needs [10,12,14-16].

With the plethora of digital technologies available today to aid in dementia caregiving, there is a need for an evidence-based, engaging, adaptable, and preference-based platform for informal caregivers of people living with dementia to aid them in identifying resources and education on dementia relevant to their and their care recipient’s needs [5,7,11,17]. Previously, our qualitative study reported that caregivers are looking for several features in such a web-based navigation and resource platform: “(1) a comprehensive database of commonly needed professional services, (2) mental health and caregiver support groups, (3) educational resources on dementia and caregiving, [and] (4) a platform that is easy to use, aesthetic, reliable, and interactive” [5]. Some current solutions that function as resource finders or navigators include the Community Resource Finder by AARP and the Alzheimer’s Association, the Alzheimer’s Navigator by the Alzheimer’s Association, and CareNav by the Family Caregiver Alliance. While these web-based tools provide databases and tips for locating resources and education on dementia and caregiving, they do not provide categorized recommendations or resources tailored to the stage of dementia of the people living with dementia and based on caregiver characteristics and preferences.

The overall performance rating of a digital app by users is greatly affected by several behavioral factors according to the theory of planned behavior, which states that an individual’s intention to use or adopt technologies is greatly influenced by their attitude toward the technologies [18,19]. Thus, the more favorably a caregiver views a technological intervention, the more likely they will be to adopt and use the product. In addition, an individual’s adoption of a technological intervention is greatly influenced by their perception of technology self-efficacy, which is defined as an individual’s confidence in applying a technology to perform a task [20-23]. The more positive an individual’s attitude and initial experience, the more likely they are to use the technology as their perceived self-efficacy increases [24]. The Mobile Application Rating Scale (MARS) is a widely used tool to test the performance of health mobile apps and digital platforms based on the functionality, design, information quality, engagement, and subjective quality of the digital apps [25].

### Objectives

To address caregivers’ needs and expectations of a web-based platform to assist them in the care of their care recipient, we developed a web-based care planning tool, Olera.care, that assists caregivers in navigating common challenges by supplying personalized recommendations and curated sets of resources (eg, care services, products, and professionals) as well as education on dementia and caregiving for people living with dementia. This pilot study aims to evaluate the functionality and usability of the initial Olera.care platform for caregivers of people living with dementia in addressing their needs with personalized education and resource matching.

## Methods

### Overview

**Figure 1:**
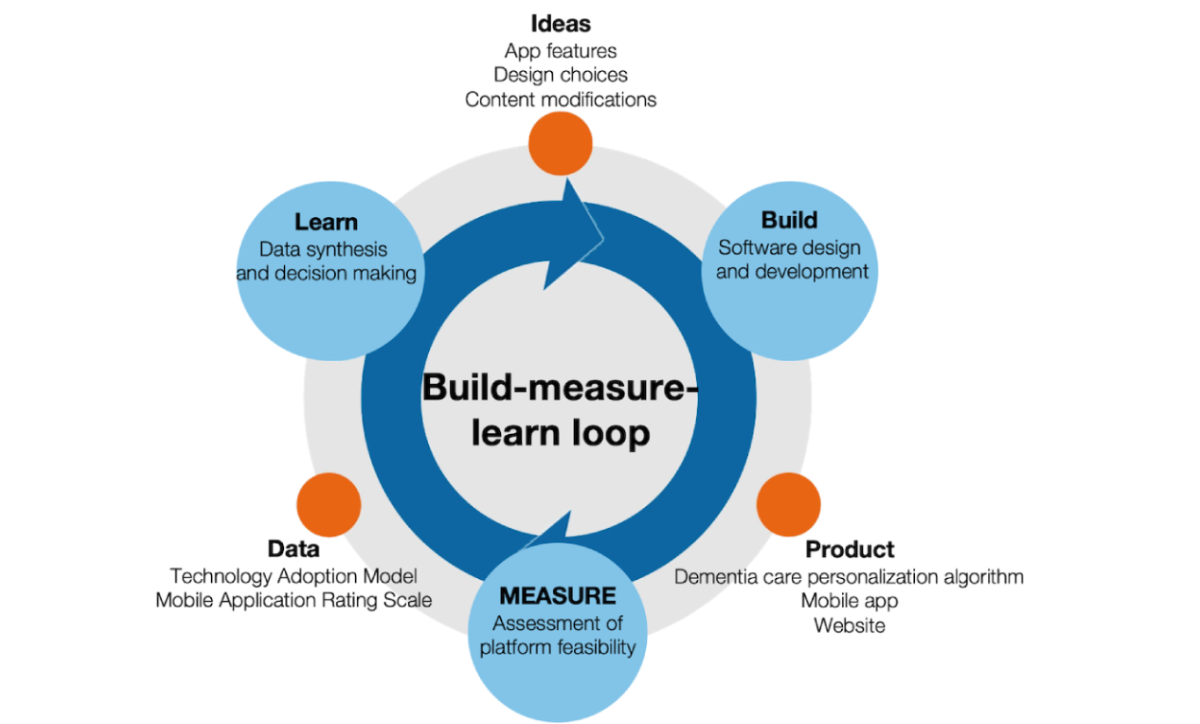
The build-measure-learn approach used to research and develop Olera.care.

This pilot study was conducted to support the development and evaluation of the usability of the Olera.care digital platform. The development and testing of the platform adopt an iterative “build-measure-learn” approach (Figure 1) that places caregivers at the forefront of design and development, ensuring that our platform continually evolves to meet their evolving needs and expectations [26]. This framework ensures that our platform iteratively evolves through an ongoing process of design and development, incorporating frequent touch points with family caregivers to assess the usability and functionality of a given prototype and align subsequent development with caregiver wishes and expectations for digital assistance technology. Participants in the study were engaged in 2 rounds of Zoom (Zoom Video Communications, Inc) or telephone interviews from January 2022 to May 2022 to (1) understand their caregiver needs, (2) engage with the platform, and (3) complete a technology survey assessment, including the modified MARS, via a Qualtrics (Qualtrics International Inc) web-based form. Each MARS item used a 5-point scale to assess the engagement, functionality, aesthetics, and information quality of the Olera.care digital platform among unpaid caregivers of people living with dementia in Texas.

### Platform Development and Testing

#### Development of a Digital Platform Capable of Providing Personalized Information on the Legal, Financial, and Estate Planning Aspects of Caregiving for Dementia, Including Information on Local Resources Most Relevant to Caregivers

**Figure 2:**
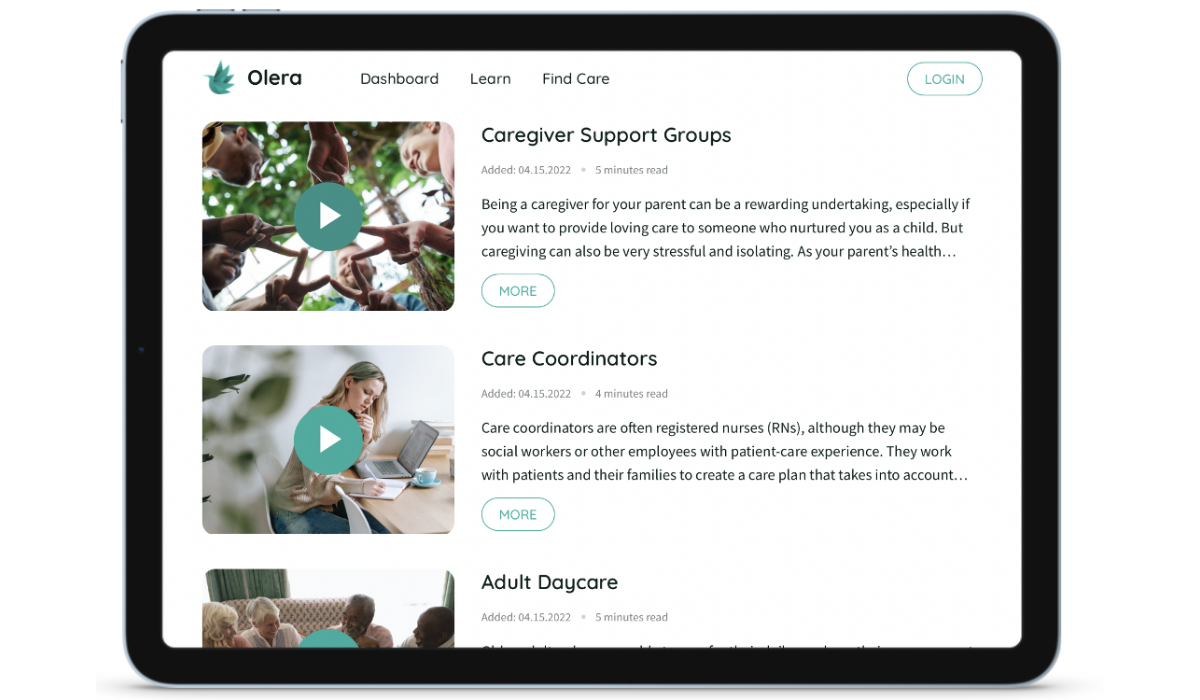
Content repository example of the Olera.care platform.

We developed a robust web application that provides tailored educational videos and articles on topics associated with the most prominent challenges and struggles that caregivers of people living with dementia face as evidenced by our previous work identifying common pain points in the caregiving journey [5]. Personalized information is curated based on answers to a caregiving questionnaire and an algorithm developed to sift through a data repository and present the most relevant information pertaining to a user’s circumstances. Our growing content repository currently hosts 66 original articles and video postings that cover various topics, including legal, financial, and estate planning. In addition to personalized education, the developed web application can present tailored listings of relevant professionals in the legal, financial, home care, older adult living, and older adult care coordination industry (Figure 2). Credentialed professionals are presented in a personalized directory that is curated based on our algorithm’s assessment of caregivers’ current professional needs, preferences, and geographic location.

#### Compilation and Management of Relevant Financial, Legal, and Estate Planning Resources Available in Our Target Area

**Figure 3:**
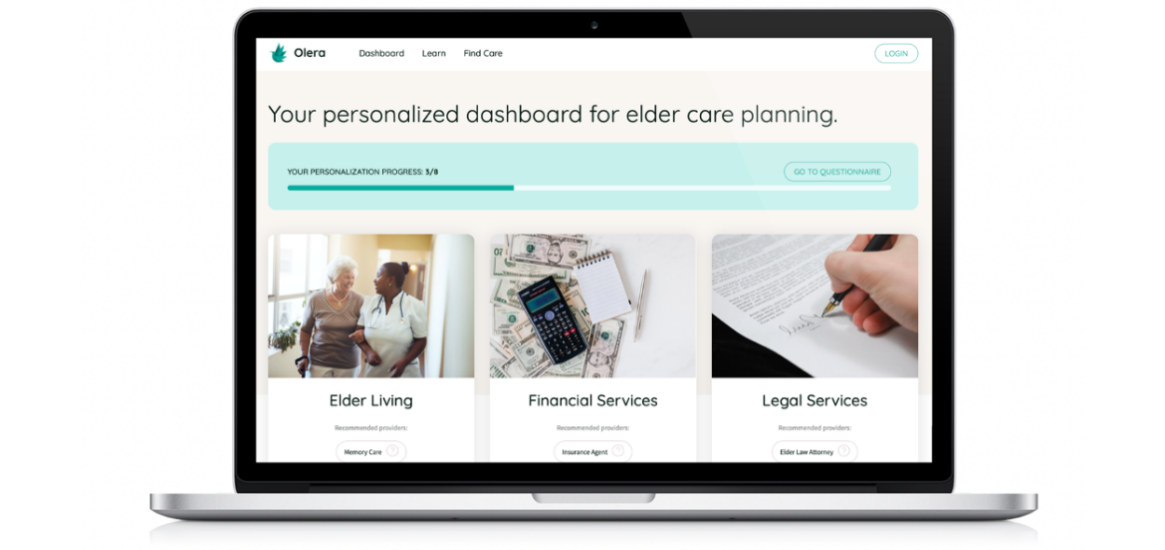
Personalized dashboard for older adult care planning on the Olera.care platform.

We have created a repository database that includes information on >22,000 professional service providers and 66 original educational articles or videos. This database is integrated with our web application’s user interface to allow users to sort through it quickly and effortlessly for information most relevant to their current needs (Figure 3). Of note, relevant service providers included in our database are certified financial planners, Medicare insurance agents, older adult law attorneys, older adult care planners, older adult housing facilities (memory care and assisted or independent living), rehabilitation centers, hospice or palliative care agencies, and in-home professional caregivers as well as home health services. These services have been included because of direct commentary noted on interviewing current dementia caregivers about their service needs when providing AD and AD-related dementia family caregiving for a loved one [5].

#### Preliminary Assessments of the Usability and Functionality of the Technology Using Techniques Such as End-User Surveys and Real-Time Monitoring

We evaluated the usability and functionality of our caregiver support platform with a pilot panel of 30 current family caregivers of persons living with dementia who met our eligibility criteria. Participants interacted with the web application in a test environment. They were asked to use the main functions and rate the usability and functionality on a modified MARS, which is a well-established instrument used as a benchmark for digital health apps. In addition, real-time monitoring during participation helped researchers identify areas for improvement in the user experience.

### Participant Eligibility and Recruitment

Participants were recruited through academic and community networks, such as the Texas A&M University Center for Community Health and Aging and the Brazos Valley Area Agency on Aging. We used both traditional and digital channels to reach out to potential participants. These included printed materials such as physical or electronic flyers, outreach emails, and social media platforms (eg, Facebook, Instagram, and Twitter) as well as engagement with web-based forums such as local caregiver support group meetings.

Individuals expressing interest in participating in the study were provided 2 options to complete the eligibility screening: participants could access a web-based form by following the recruitment materials’ QR code or web link; alternatively, they could contact the Olera.care team via telephone to obtain information about the study and take the eligibility screening survey over the telephone.

The eligibility screening survey collected relevant information to examine whether interested individuals met the following inclusion criteria: (1) be aged ≥18 years; (2) be a nonpaid caregiver of a person living with dementia; (3) be the adult child, spouse or partner, other family member, or legal guardian of a person living with dementia; (4) be engaged in making legal or financial, older adult living, or medical decisions for a person living with dementia; (5) be seeking older adult care services in Texas; and (6) have access to a smartphone or computer with internet access.

After the eligibility screening process, participants who met the inclusion criteria were asked to provide their full name, contact information, preferred mode of contact (ie, telephone or email), and preferred day and time for subsequent outreach by the research team.

### Ethical Considerations

Ethics approval was obtained from the Texas A&M University Institutional Review Board (2021-0943D). The study personnel asked all participants to provide electronic informed consent in the screening survey. Upon the completion of the eligibility screening survey, participants were presented with an informed consent document. This document provided instructions on how to convey their consent to participate in the study and their willingness to be recorded and followed up via the Qualtrics platform. The consent form covered important information, including the rationale for inclusion; the research objectives; the voluntariness of participation with the option to withdraw at any point; the anticipated participation duration and procedures; the potential risks, benefits, and costs of participation; and how participant confidentiality would be protected.

### Assessments and Measurements

#### Evaluating the Quality of the Olera.care Platform Using the MARS

We used the MARS as a robust assessment tool to evaluate the quality of the Olera.care platform among the caregiver participants. The MARS provides a multidimensional assessment of the engagement, functionality, aesthetics, information quality, and overall subjective quality of the Olera.care platform. To tailor the assessment to our study’s specific context, we selected 13 items from the MARS questionnaire that were directly relevant to the Olera.care platform. Our methodology for adapting the MARS questionnaire was 2-pronged: first, we evaluated and retained items based on their relevance to our platform’s functions, omitting nonapplicable elements such as gestural design; and second, we modified the wording of the retained items to better reflect our platform’s unique features. These items were adapted and modified for our assessment objectives while retaining the original item classification across the 5 dimensions. The modified items and responses, while maintaining the integrity of the MARS, ensure an effective evaluation of our digital platform. Participants provided ratings for each MARS item using a 5-point scale (1=inadequate, 2=poor, 3=acceptable, 4=good, and 5=excellent), with each response tailored to the content of the respective item. An overall subjective quality rating of ≥3.6 was set as the threshold to indicate good usability and quality of the Olera.care platform according to past literature [27], allowing us to effectively report the platform’s overall quality from assessment among the caregiver users.

#### Assessing Willingness, Self-Efficacy, and Communication Preferences in Technology Adoption

To understand the willingness and preference for technology use, we further assessed participants’ intention to use the Olera.care platform, self-efficacy for using the technology, and preferences for web-based communication and platform format. The participants were asked whether they would like to use the technology in the future, with the response options being “yes,” “maybe,” and “no.” They were also asked about their confidence level in using the technology, with the response options being “uncertain,” “neither certain nor uncertain,” “somewhat confident,” and “very confident.” In addition, participants were asked about their preference for anonymity when seeking information on the internet and whether they would consider sharing personal information for receiving individualized answers. The net promoter score, which was created in 2003 and has been used in a variety of industries such as insurance, technology services, communications, and health care [28], was used to evaluate the willingness of participants and provide insights for user experience management. Responses on a scale ranging from 0=strongly disagree to 10=strongly agree were used. Scores from 0 to 6 were classified as *detractor*, scores of 7 and 8 were categorized as *passive*, and scores of 9 and 10 were designated as *promoter*. Participants were also asked whether they preferred a website-based format or a mobile app format.

#### Assessing the Use of, and Interests in, Older Adults Care Services Among Caregivers

To gain a deeper understanding of our targeted population’s preferences and needs for planning older adult care and to continually enhance and optimize our platform, we assessed the level of interests in older adult care services among caregivers. Caregivers were presented with 21 types of older adult care services (eg, home health, hospice care, memory care, caregiver support group, and assisted living), and they were asked to select ≥1 of the following responses: “currently using,” “have used before,” “would like to learn more,” and “would never use.” This assessment allowed us to gather valuable insights into caregivers’ engagement and interests in various older adult care options, informing our efforts to better serve their needs.

#### Sociodemographic and Caregiving Characteristics of Caregivers

We collected the sociodemographic and caregiving characteristics of participants to understand how representative our study population was for the Texas caregiver profile and whether the major platform evaluation outcomes differ by the background characteristics of participants. The caregiver characteristics collected included age, sex, race, ethnicity, the highest level of education completed, employment status, general financial status, caregiving role, and length of providing care.

### Analysis

Descriptive statistics were used to describe the engagement, functionality, aesthetics, information quality, and subjective quality of the platform and caregiver characteristics and responses. Mean scores and SDs were calculated for each MARS item. We conducted 2-sample 2-tailed *t* tests to compare the differences in the major MARS evaluation scores by caregiver characteristics. All analyses were conducted using Stata (version 17.0; StataCorp LLC).

## Results

### Participants’ Characteristics

**Figure 4:**
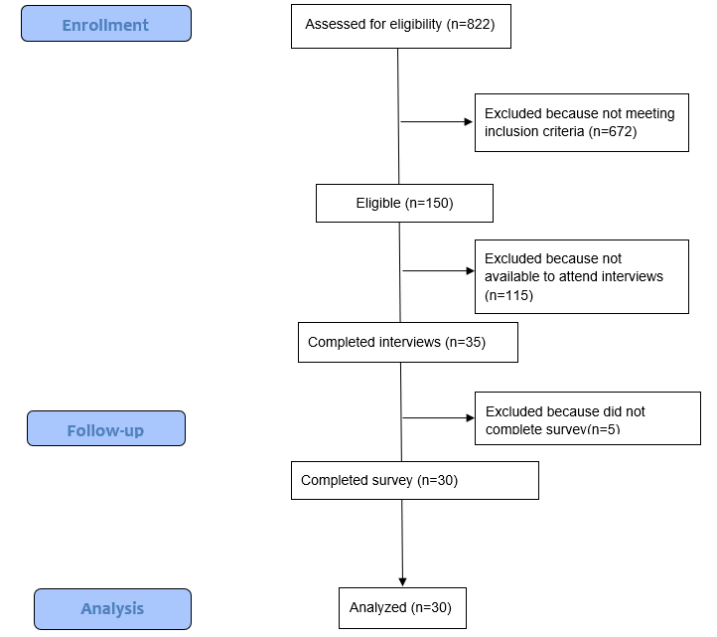
Study flow diagram.

Of the initial 822 respondents who completed the prescreening surveys, 150 (18.2%) met the eligibility criteria, of whom, after excluding 115 (76.7%) individuals for not being available to attend interviews, 35 (23.3%) were enrolled into the study. Of these 35 enrolled individuals, 30 (86%) interacted with the platform and completed the study survey of technology evaluation and caregiving needs (Figure 4). The sociodemographic and caregiving characteristics of participants are summarized in Table 1.

Of the 30 participants, the majority were aged ≥50 years (n=25, 83%), women (n=23, 77%) , White (n=25, 83%), non-Hispanic (n=27, 90%), had bachelor’s or graduate degrees (n=22, 73%), and were employed for wages (n=12, 40%) or retired (n=12, 40%). Financially, 67% (20/30) had surplus funds at the end of each month, while 30% (9/30) just about managed to meet their expenses or faced deficits. Most of the participants were recruited via email invitations (13/30, 43%) or web-based advertisements (11/30, 37%), with some also recruited through in-person presentations and personal connections (5/30, 17%). In terms of caregiving characteristics, the majority of the participants identified themselves as primary caregivers (20/30, 67%), reported to have provided care for at least 1 year (28/30, 93%), and dedicated at least 20 hours weekly to caregiving in the past 3 months (21/30, 70%).

**Table 1:** Sociodemographic and caregiving characteristics of participants (n=30).

Characteristics			Participants, n (%)
**Age (y)**			
	35-49	5 (17)	
	50-64	12 (40)	
	≥65	13 (43)	
**Sex**			
	Male	7 (23)	
	Female	23 (77)	
**Race**			
	American Indian or Alaska Native	1 (3)	
	Asian, Native Hawaiian, or Pacific Islander	1 (3)	
	Black or African American	2 (7)	
	White	25 (83)	
	Multiracial	1 (3)	
**Ethnicity**			
	Spanish, Hispanic, or Latinx origin or descent	3 (10)	
	Other	27 (90)	
**Highest level of education completed**			
	Some college but no degree	5 (17)	
	Associate degree	3 (10)	
	Bachelor’s degree	8 (27)	
	Graduate degree	14 (47)	
**Employment status**			
	Employed for wages	12 (40)	
	Homemaker or self-employed	4 (13)	
	Unemployed or unable to work	2 (7)	
	Retired	12 (40)	
**General financial status at the end of the month**			
	End up with some money left over	20 (67)	
	Have just enough to make ends meet	7 (23)	
	Not have enough money to make ends meet	2 (7)	
	Do not know	1 (3)	
**Caregiving role in providing care for ≥1 adults aged >50 y**			
	Participant is the primary caregiver	20 (67)	
	Someone else is the primary caregiver	4 (14)	
	The participant shared caregiving responsibilities about equally with someone else	5 (17)	
	Unknown	1 (3)	
**How long has the participant been providing care or assistance for the care recipients? (y)**			
	0.5 to <1	2 (7)	
	1 to <5	14 (47)	
	5 to <10	8 (27)	
	≥10	6 (20)	
**Over the past 3 months, about how many h/wk has the participant provided some form of care for ≥1 adults aged >50 y?**			
	<20	9 (30)	
	20-40	9 (30)	
	>40	12 (40)	
**Recruitment channel**			
	Online advertisement (Facebook, LinkedIn, etc)	11 (37)	
	Email invitation	13 (43)	
	In-person presentation	2 (7)	
	Personal connection	3 (10)	
	Other	1 (3)	

### Quality Assessment of the Olera.care Platform

#### Overview

The descriptive statistics of the Olera.care platform evaluation by the MARS dimensions and items are presented in Table 2. The mean overall satisfaction rating of the Olera.care platform among the participants was 4.57 (SD 0.57) of 5. Mean scores for engagement, functionality, aesthetics, and information quality were 4.10 (SD 0.61), 4.46 (SD 0.44), 4.58 (SD 0.53), and 4.76 (SD 0.44), respectively.

**Table 2:** Modified Mobile Application Rating Scale items and responses of participants.

Dimensions, items, questions, and response items				Participants, n (%)		Score, mean (SD)	
**Engagement**							4.10 (0.61)
	**Entertainment: compared to other older adult care finders, websites, or alternative resources for older adult care information you may have used, do you think this app is fun to use?**						3.67 (0.80)
		Highly entertaining	5 (17)				
		Fun	11 (37)				
		OK, fun enough	13 (43)				
		Mostly dull	1 (3)				
		Dull	0 (0)				
	**Interestingness: compared to other older adult care finders, websites, or alternative resources for older adult care information you may have used, does this app present its content in an interesting way?**						4.53 (0.57)
		Very interesting	17 (57)				
		Interesting	12 (40)				
		Slightly interesting	1 (3)				
		Mostly uninteresting	0 (0)				
		Not interesting	0 (0)				
**Functionality**							4.46 (0.44)
	**Performance: how well do the app’s features, components, and buttons work?**						4.57 (0.57)
		Works perfectly	18 (60)				
		Very functional	11 (37)				
		App works overall	1 (3)				
		Some functions work	0 (0)				
		App is broken	0 (0)				
	**Ease of use: how easy is it to learn how to use the app?**						4.57 (0.50)
		Very simple	17 (57)				
		Easy to learn	13 (43)				
		Usable	0 (0)				
		Somewhat confusing	0 (0)				
		Very confusing	0 (0)				
	**Navigation: does moving between pages make sense?**						4.28 (0.59)
		Moving between pages is perfectly logical, easy, clear and intuitive	10 (33)				
		Moving between pages is easy to understand and navigate	17 (57)				
		Moving between pages is understandable after some time and effort	2 (7)				
		Moving between pages is understandable after a lot of time and effort	0 (0)				
		Moving between pages is difficult	0 (0)				
		Missing	1 (3)				
**Aesthetics**							4.58 (0.53)
	**Visual appeal: how good does the app look?**						4.53 (0.63)
		Very visually appealing	18 (60)				
		High-level visual appeal	10 (33)				
		Some visual appeal	2 (7)				
		Little visual appeal	0 (0)				
		No visual appeal	0 (0)				
	**Graphics: how high is the quality of graphics, buttons, and content?**						4.53 (0.63)
		Very high quality	18 (60)				
		High quality	10 (33)				
		Moderate quality	2 (7)				
		Low quality	0 (0)				
		Very poor quality	0 (0)				
	**Layout: how would you rate the design? Are the arrangement and size of buttons and content on the screen appropriate?**						4.67 (0.61)
		Very professional	22 (73)				
		Mostly professional	6 (20)				
		Satisfactory	2 (7)				
		Bad design	0 (0)				
		Very bad design	0 (0)				
**Information**							4.76 (0.44)
	**Accuracy of app description: after reviewing the home page, does the app contain what is advertised and described?**						4.79 (0.41)
		Highly accurate	23 (77)				
		Mostly accurate	6 (20)				
		Somewhat accurate	0 (0)				
		Slightly misleading	0 (0)				
		Very misleading	0 (0)				
		Missing	1 (3)				
	**Quality of information: is the content in the app relevant to helping with older adult care planning?**						4.76 (0.44)
		Highly relevant	22 (73)				
		Relevant	7 (23)				
		Moderately relevant	0 (0)				
		Barley relevant	0 (0)				
		Irrelevant content	0 (0)				
		Missing	1 (3)				
	**Quality of visual information: are images, videos, and graphics clear and easily understandable?**						4.73 (0.45)
		Perfectly clear	22 (73)				
		Mostly clear	8 (27)				
		Somewhat clear	0 (0)				
		Mostly unclear	0 (0)				
		Completely unclear	0 (0)				
**Subjective quality**							4.15 (0.51)
	**Stimulates repeat use: how many times do you think you would use this app in the next 12 months if it was relevant to you?**						3.77 (0.86)
		>50	6 (20)				
		10-50	13 (43)				
		3-10	9 (30)				
		1-2	2 (7)				
		None	0 (0)				
	**Worth recommending: would you recommend this app to people who might benefit from it?**						4.27 (0.14)
		I would recommend this app to everyone	13 (43)				
		There are many people I would recommend this app to	12 (40)				
		There are several people I would recommend it to	5 (17)				
		There are a few people I would recommend this app to	0 (0)				
		I would not recommend this app to anyone	0 (0)				
	**Overall satisfaction rating: what is your overall star rating of the app? (1 star=poor; 5 stars=excellent)**						4.57 (0.57)
		5	17 (57)				
		4	10 (33)				
		3	1 (3)				
		2	0 (0)				
		1	0 (0)				
		Missing	2 (7)				

#### Engagement

Participants reported high levels of engagement with the Olera.care platform, with 97% (29/30) describing it highly entertaining or fun or fun enough, and 97% (29/30) expressing that it was interesting to interact with.

#### Functionality

In terms of functionality, the majority of the participants assessed the Olera.care platform positively, with 60% (18/30) perceiving it as working perfectly and 37% (11/30) rating it as very functional. Moreover, 57% (17/30) found it very simple to learn to use, and 43% (13/30) considered it easy to learn. An impressive 90% (27/30) of the participants reported that it was easy to navigate between pages.

#### Aesthetics

The aesthetics of the platform received favorable feedback from participants, with 93% (28/30) expressing that it was visually appealing, highlighting the high quality of graphics, buttons, and content. The design and layout of the content were described as professional.

#### Information

In terms of information quality, nearly all participants (29/30, 97%) noted that the app contained relevant and clear information. A substantial 73% (22/30) found the information highly relevant for older adult care planning, while 23% (7/30) considered it relevant. Visual information was deemed perfectly clear by 73% (22/30) of the participants and mostly clear by 27% (8/30).

#### Subjective Quality

Participants expressed a strong inclination to use the app in in the next 12 months, with 20% (6/30) planning to use it >50 times and 43% (13/30) aiming to use it between 10 and 50 times. Importantly, all participants indicated their willingness to recommend the app to those who may benefit from it. In terms of overall satisfaction, most of the participants rated the Olera.care platform positively, with 57% (17/30) rating it as excellent and 33% (10/30) as very good.

### Intention to Use the Olera.care Platform, Self-Efficacy of Technology Use, and Platform Preference

In this study, a substantial proportion of the participants expressed a positive intention to use the Olera.care platform (Table 3), with 90% (27/30) indicating a definite willingness (“yes”), while the remaining 10% (3/30) expressed a more tentative interest (“maybe”). In terms of self-efficacy in using technology, 40% (12/30) of the participants reported feeling “very confident,” 57% (17/30) indicated a moderate level of confidence (“somewhat confident”), and 3% (1/30) expressed uncertainty. In the context of online communication with a representative, 70% (21/30) of the participants favored remaining anonymous before sharing contact information. Furthermore, 43% (13/30) of the participants expressed willingness to share personal information to receive personalized assistance. In terms of platform preference, most of the participants (22/30, 73%) preferred the computer browser format, 10% (3/30) preferred to use a mobile app, and 17% (5/30) did not have a preference. This preference for computer browser format directly ties into overall user satisfaction, which is a critical determinant in the net promoter score categorization. Focusing on optimizing this platform could lead to higher user satisfaction and thus more promoters.

**Table 3:** Intention to use, self-efficacy of use, and preferences for communication and platform.

Questions and response items				Participants, n (%)
**Intention to use the app**				
	**Would you use this app?**			
		Yes	27 (90)	
		Maybe	3 (10)	
		No	0 (0)	
**Self-efficacy to use technology**				
	**When it comes to your confidence in the use of technology, which of the following best describes you?**			
		Uncertain	1 (3)	
		Neither certain nor uncertain	0 (0)	
		Somewhat confident	17 (57)	
		Very confident	12 (40)	
**Preferences when asking a representative**				
	**When researching older adult services on the web, I’d appreciate the ability to ask a representative a question anonymously before sharing my contact information (0=strongly disagree; 10=strongly agree).**			
		9-10 (promoter)	21 (70)	
		7-8 (passive)	3 (10)	
		0-6 (detractor)	6 (20)	
	**When researching older adult services online, I would consider sharing personal information (contact information, etc) with a representative to better determine my loved one’s fit for the service (0=strongly disagree; 10=strongly agree).**			
		9-10 (promoter)	13 (43)	
		7-8 (passive)	6 (20)	
		0-6 (detractor)	11 (36)	
**Preference for platform**				
	**When searching online for information on older adult care planning, would you prefer to use a computer browser or download a mobile app?**			
		Computer browser	22 (73)	
		Mobile app I can download to my phone	3 (10)	
		No preference	5 (17)	

### Caregivers’ Use of, and Interests in, Older Adult Care Services

We also examined the current and past use of, as well as interests in, different types of older adult care services among the caregiver participants (Table 4). Among the participants, 63% (19/30) were currently using medical home health services, 33% (10/30) used hospice care, and 30% (9/30) employed certified financial planners. In addition, 27% (8/30) relied on house maintenance service, and 23% (7/30) engaged insurance agents and older adult law attorneys and participated in public and free older adult programs. Memory care, nonmedical home aid, and adult day care each had a 20% (6/30) use rate, while services such as assisted living, independent living, transportation, skilled nursing, medical providers, and rehabilitation facilities were used by 7% (2/30) to 13% (4/30) of the participants. Older adult living referral agents, caregiver support groups, and yard services were used by 3% (1/30) of the participants. The results suggest the diverse range of older adult care services currently being used, with medical home health being the most used service.

**Table 4:** Caregivers’ use of, and interests in, older adult care services (n=30).

Older adult care services		Participants, n (%)
**Using currently**		
	Home health	19 (63)
	Hospice care	10 (33)
	Certified financial planner	9 (30)
	House maintenance	8 (27)
	Insurance agent	7 (23)
	Older adult law attorney	7 (23)
	Public and free older adult programs	7 (23)
	Adult day care	6 (20)
	Memory care	6 (20)
	Professional home caregivers	6 (20)
**Have used before**		
	Professional home caregivers	13 (43)
	Older adult law attorney	10 (33)
	Rehabilitation facility	10 (33)
	Insurance agent	9 (30)
	Certified financial planner	7 (23)
	Older adult living referral agent	7 (23)
	Hospice care	7 (23)
	Transportation services	7 (23)
	Skilled nursing	6 (20)
	Home health	6 (20)
	Public and free older adult programs	6 (20)
**Would never use**		
	Yard services	8 (27)
	Certified financial planner	7 (23)
	House maintenance service	7 (23)
	Care manager	7 (23)
	Insurance agent	5 (17)
	Independent living	5 (17)
	Assisted living	4 (13)
	Meal service	4 (13)
	Memory care	3 (10)
	Rehabilitation facility	3 (10)
	Older adult living referral agent	3 (10)
**Would like to learn more**		
	Caregiver support group	24 (80)
	Medical providers	23 (77)
	Memory care	19 (63)
	Meal service	19 (63)
	Adult day care	19 (63)
	Older adult living referral agent	18 (60)
	Transportation services	18 (60)
	Care manager	18 (60)
	Skilled nursing	17 (57)
	Assisted living	17 (57)
	Public and free older adult programs	17 (57)

Regarding past use, 43% (13/30) had previously used nonmedical home aid, and 33% (10/30) had engaged older adult law attorneys and used rehabilitation facilities. Insurance agents were consulted by 30% (9/30) of the participants, and 23% (7/30) had used certified financial planners and older adult living referral agents, as well as hospice care and transportation services. Skilled nursing, medical home health, and public and free older adult programs were previously used by 20% (6/30) of the participants, while independent living and adult day care had a use rate of 17% (5/30). Memory care, assisted living, meal services, and yard services were used by 13% (4/30), while house maintenance, care managers, and medical providers were used by 7% (2/30). Caregiver support groups were attended by 3% (1/30) of the participants.

In terms of preferences for further exploration, 80% (24/30) expressed interest in learning more about care support groups, while 77% (23/30) were interested in understanding medical providers better. In addition, 63% (19/30) showed interest in memory care, meal services, and adult day care. Furthermore, 60% (18/30) were interested in older adult living referral agents, transportation services, and care managers. An additional 57% (17/30) desired to learn more about skilled nursing, assisted living, and public and free older adult programs. Interestingly, all older adult care services were mentioned as areas of interest by participants.

For services that would never be considered, some of the participants (18/30, 60%) indicated reluctance toward specific services, with yard services (8/30, 27%), certified financial planners (7/30, 23%), and house maintenance (7/30, 23%) being among those mentioned, while none expressed a definitive refusal to consider nonmedical home aid or hospice care.

### Platform Feature Evaluation Results by Participants’ Characteristics

We conducted 2-sample *t* tests to assess variances in platform feature evaluation results based on caregivers’ characteristics, providing mean scores and SDs for reporting (Multimedia Appendix 1). The evaluation results for the Olera.care platform, spanning engagement, functionality, aesthetics, information quality, and overall satisfaction, were notably consistent across all caregiver groups. However, a statistically significant difference (*P*=.02) was observed in the functionality evaluation scores, with caregivers dedicating at least 20 hours to care (mean 4.6, SD 0.4) rating it higher than those providing less care (mean 4.2, SD 0.5). In addition, caregivers with <5 years of caregiving experience reported significantly higher evaluation scores for aesthetics (mean 4.7, SD 0.4 vs mean 4.3, SD 0.7; *P*=.04) and information quality (mean 4.8, SD 0.2 vs mean 4.6, SD 0.3; *P*=.03) compared to those with a minimum of 5 years of caregiving experience.

## Discussion

### Principal Findings

This research lays the foundation for the development of digital tools tailored to the needs of caregivers. The principal findings involve the quality and usability of the Olera.care platform, a web-based care planning tool designed to assist caregivers of people living with dementia in addressing their legal and financial needs and enable them to access functional care services. The results suggest that the Olera.care web tool is a practical, engaging, easy-to-use, visually appealing, and informative digital platform designed to provide resources that address common challenges faced by family caregivers of people living with dementia [5]. The study assessed caregivers’ intentions to use the Olera.care platform, their expectations for caregiving educational content, and their preferences for web-based information delivery. These aspects are crucial in our iterative build-measure-learn framework of research and development, which underpins our commitment to caregiver-centric product design.

The results indicate that the tested Olera.care web tool can distinguish itself not only in terms of practicality and user-friendliness but also in the quality of its content and its degree of personalization. We acknowledge existing solutions such as the Community Resource Finder by AARP and the Alzheimer’s Association, the Alzheimer’s Navigator by the Alzheimer’s Association, and CareNav by the Family Caregiver Alliance. These platforms offer valuable databases and guidance for dementia caregiving. However, our Olera.care platform differentiates itself by providing recommendations and resources that are not only categorized but also personalized and tailored to the caregiver’s specific characteristics and preferences. This unique aspect of Olera.care addresses a gap in current offerings and stands in contrast to many currently available web-based information tools such as static web pages, resource directories, or learning modules, which can be inadequate in addressing certain needs due to their lack of user engagement, personalization, relevance, and adoptability [29,30]. By contrast, the Olera.care platform attempts to involve users in the design of the platform and address these issues effectively, and the platform stands out for its interactivity, visual appeal, personalization capabilities, and informative content, making it a valuable resource for family caregivers of people living with dementia.

One noteworthy finding is that participants who devoted more weekly hours to caregiving and had limited cumulative caregiving experience tended to rate the Olera.care platform more favorably. This suggests that the platform offers specific support and benefits to caregivers with heavier caregiving workloads and those with limited prior caregiving experience. This insight underscores the importance of tailoring digital tools to the specific needs of caregivers in different situations, considering their experience and time commitment [29,30]. Such findings are integral to the build-measure-learn framework, guiding the iterative development of the platform to better align with the specific needs of caregivers. Furthermore, the relevance of the Olera.care platform is heightened in the context of the COVID-19 pandemic, which significantly disrupted family caregiving arrangements, as evidenced by more than half of these arrangements being affected [31]. This disruption led to heightened psychological burdens on caregivers, including increased depression, anxiety, and loneliness [31,32]. The pandemic also exacerbated the shortage of professional caregivers, further challenging the support systems for older adult care. Studies highlight the increased stress levels among caregivers, particularly those caring for individuals with severe dementia [33], and the overall strain on mental health resources for both caregivers and patients [34]. With the increasing social and support needs of caregivers, internet-based tools are crucial to help caregivers to access information and gain support [35].

Another significant finding pertains to the use of, and demand for, older adult care services. Many caregivers reported using or intending to use services such as home health, hospice care, insurance agents, older adult law attorneys, and financial planners. However, there is notable interest in exploring other services, such as caregiver support groups, medical providers, skilled nursing, memory care, public and free older adult programs, meal services, adult day care, and various older adult living alternatives. The data indicate that these services are often underused, potentially leading to unmet needs among older adults and their caregivers [36]. This underuse may stem from a lack of awareness about the availability of older adult care services among caregivers [37]. This insight has prompted a shift in our database curation strategy, and we are focusing now on underused yet high-demand care services and programs to make them more readily available to caregivers through our platform.

The high engagement, functionality, aesthetics, and information quality of the Olera.care digital platform can be attributed to several underlying principles and strategies. First, the platform’s unique approach of involving caregivers in the design process has proven highly effective. We demonstrated that technology interventions developed with input from the target population will increase overall satisfaction with, and preference for, the product. Second, the curated content and resources of the Olera.care platform, informed by leading experts, have been rated highly relevant by the participants, with 73% (22/30) reporting the content as highly relevant with a mean MARS score of 4.76 (SD 0.44) of 5. This demonstrates the significance of expert guidance in creating a resource that resonates with the target audience [26]. The curated content and resources on the Olera.care platform can be further improved through the incorporation of artificial intelligence and large language models, allowing for improved and personalized recommendations based on an existing recommendation system [38]. Large language models can process large data sets across numerous relevant variables (eg, specific needs, geographic location, and financial constraints) to provide the most appropriate care solutions. To increase the accuracy of the large language model, the data would have to undergo rigorous quality control and standardization. Industry experts that we are currently working with to inform our curated content could also validate the model through feedback on its accuracy and quality. The model’s recommendations can also be consistently improved through the input of new data, further increasing the accuracy of its recommendations.

### Limitations and Future Research and Practice

It is important to acknowledge the limitations of this pilot study, which we view as opportunities for further learning and refinement within our build-measure-learn framework. First, the small sample size and lack of racial and ethnic diversity among the participants may limit the generalizability of the findings, pushing us to expand our research scope. The participants in this study were also mostly technology savvy and well educated. However, the demographic characteristics of the caregiver participants in this study aligned well with the caregiver profile in the United States (ie, the majority were women: 23/30, 77%; aged ≥50 years: 25/30, 83%; and non-Hispanic White: 25/30, 83%) [39]. The study populations also represented some diversity in financial levels and caregiving experiences. Future research should aim to include a more racially and ethnically diverse study population to ensure a broader representation of caregiver experiences and preferences. Second, all acceptability and usability metrics in this study were self-reported, which could introduce self-report bias. However, we used a validated tool, the MARS [25], which was adapted according to platform features. Third, given the limited sample size and insufficient statistical power in the subgroup analyses, both significant and nonsignificant *t* test results should be interpreted cautiously. Furthermore, the study’s short duration of interaction with the digital platform also limits our understanding of the platform’s long-term usability and usefulness for caregivers of people living with dementia.

A future study should be conducted with a larger and more diverse group of caregivers, allowing for a more comprehensive assessment of the platform’s long-term perceived usability and ease of use, thus enabling us to continually learn and refine the platform. For future practice, we are focused on enhancing the accessibility and visibility of our web application for family caregivers by integrating it into the existing care delivery framework through strategic digital marketing, primarily using organic search engine optimization. This strategy ensures that our platform aligns with user search behaviors, making it easily discoverable by those in need of caregiving resources. Simultaneously, we are committed to maintaining universal accessibility, opting for a broader reach via effective search engine optimization strategies over direct integration into health plans. Our platform, designed more as a comprehensive digital health tool than a conventional medical device, provides holistic care planning support for family caregivers. Financially, we have chosen a sustainable business-to-business revenue model, focusing on advertising and commission-based referral fees from businesses serving our caregiver community, which allows us to offer our services free of charge to families. This approach is underpinned by the older adult care industry’s potential for low-cost client acquisition, enabling us to provide much-needed support to families without financial burden and ensuring that our platform remains accessible to all caregivers in need.

### Conclusions

The Olera.care platform, characterized by its practicality, interactivity, ease of use, visual appeal, and informativeness, shows promise as a valuable tool for dementia caregivers. With the pilot group of caregivers’ engagement and feedback, the platform provides tailored support to meet the specific challenges of dementia caregiving. Future development and research are essential to enhance the platform and comprehensively evaluate its efficacy in supporting caregivers and alleviating caregiving burdens across broader and more diverse populations.

## Appendix

Multimedia Appendix 1 Results of the 2-sample 2-tailed t tests for platform feature evaluation by participants’ characteristics.

## Abbreviations

AD: Alzheimer disease
MARS: Mobile Application Rating Scale

## References

[1] (2023). 2023 Alzheimer's disease facts and figures. Alzheimers Dement.

[2] Radue R, Walaszek A, Asthana S (2019). Neuropsychiatric symptoms in dementia. Handb Clin Neurol.

[3] Wolff JL, Spillman BC, Freedman VA, Kasper JD (2016). A national profile of family and unpaid caregivers who assist older adults with health care activities. JAMA Intern Med.

[4] Plöthner M, Schmidt K, de Jong L, Zeidler J, Damm K (2019). Needs and preferences of informal caregivers regarding outpatient care for the elderly: a systematic literature review. BMC Geriatr.

[5] Fan Q, DuBose L, Ory MG, Lee S, Hoang M, Vennatt J, Kew CL, Doyle D, Falohun T (2023). Financial, legal, and functional challenges of providing care for people living with dementia and needs for a digital platform: interview study among family caregivers. JMIR Aging.

[6] Connors MH, Seeher K, Teixeira-Pinto A, Woodward M, Ames D, Brodaty H (2020). Dementia and caregiver burden: a three-year longitudinal study. Int J Geriatr Psychiatry.

[7] Block L, Gilmore-Bykovskyi A, Jolliff A, Mullen S, Werner NE (2020). Exploring dementia family caregivers' everyday use and appraisal of technological supports. Geriatr Nurs.

[8] Bhargava Y, Baths V (2022). Technology for dementia care: benefits, opportunities and concerns. J Glob Health Rep.

[9] Wójcik D, Szczechowiak K, Konopka P, Owczarek M, Kuzia A, Rydlewska-Liszkowska I, Pikala M (2021). Informal dementia caregivers: current technology use and acceptance of technology in care. Int J Environ Res Public Health.

[10] Astell AJ, Bouranis N, Hoey J, Lindauer A, Mihailidis A, Nugent C, Robillard JM, TechnologyDementia Professional Interest Area ... (2019). Technology and dementia: the future is now. Dement Geriatr Cogn Disord.

[11] Kinchin I, Edwards L, Adrion E, Chen Y, Ashour A, Leroi I, Brugulat-Serrat A, Phillips J, Masterson F, Kochovska S (2022). Care partner needs of people with neurodegenerative disorders: what are the needs, and how well do the current assessment tools capture these needs? A systematic meta-review. Int J Geriatr Psychiatry.

[12] Lorenz K, Freddolino PP, Comas-Herrera A, Knapp M, Damant J (2019). Technology-based tools and services for people with dementia and carers: mapping technology onto the dementia care pathway. Dementia.

[13] McCabe M, You E, Tatangelo G (2016). Hearing their voice: a systematic review of dementia family caregivers' needs. Gerontologist.

[14] Garcia-Ptacek S, Dahlrup B, Edlund AK, Wijk H, Eriksdotter M (2019). The caregiving phenomenon and caregiver participation in dementia. Scand J Caring Sci.

[15] Ambegaonkar A, Ritchie C, de la Fuente Garcia S (2021). The use of mobile applications as communication aids for people with dementia: opportunities and limitations. J Alzheimers Dis Rep.

[16] Martínez-Alcalá CI, Pliego-Pastrana P, Rosales-Lagarde A, Lopez-Noguerola JS, Molina-Trinidad EM (2016). Information and communication technologies in the care of the elderly: systematic review of applications aimed at patients with dementia and caregivers. JMIR Rehabil Assist Technol.

[17] Chelberg GR, Neuhaus M, Mothershaw A, Mahoney R, Caffery LJ (2022). Mobile apps for dementia awareness, support, and prevention - review and evaluation. Disabil Rehabil.

[18] Ajzen I (1991). The theory of planned behavior. Organ Behav Hum Decis Process.

[19] Acikgoz F, Elwalda A, De Oliveira MJ (2023). Curiosity on cutting-edge technology via theory of planned behavior and diffusion of innovation theory. Int J Inf Manage Data Insights.

[20] Bandura A (1977). Self-efficacy: toward a unifying theory of behavioral change. Psychol Rev.

[21] An F, Xi L, Yu J, Zhang M (2022). Relationship between technology acceptance and self-directed learning: mediation role of positive emotions and technological self-efficacy. Sustainability.

[22] Rahman MS, Ko M, Warren J, Carpenter D (2016). Healthcare Technology Self-Efficacy (HTSE) and its influence on individual attitude: An empirical study. Comput Human Behav.

[23] Pan X (2020). Technology acceptance, technological self-efficacy, and attitude toward technology-based self-directed learning: learning motivation as a mediator. Front Psychol.

[24] Laganà L, Oliver T, Ainsworth A, Edwards M (2011). Enhancing computer self-efficacy and attitudes in multi-ethnic older adults: a randomised controlled study. Ageing Soc.

[25] Stoyanov SR, Hides L, Kavanagh DJ, Zelenko O, Tjondronegoro D, Mani M (2015). Mobile app rating scale: a new tool for assessing the quality of health mobile apps. JMIR Mhealth Uhealth.

[26] Blank S (2020). The Four Steps to the Epiphany: Successful Strategies for Products That Win.

[27] Mandracchia F, Llauradó E, Tarro L, Valls RM, Solà R (2020). Mobile phone apps for food allergies or intolerances in app stores: systematic search and quality assessment using the mobile app rating scale (MARS). JMIR Mhealth Uhealth.

[28] Hamilton DF, Lane JV, Gaston P, Patton JT, MacDonald DJ, Simpson AH, Howie CR (2014). Assessing treatment outcomes using a single question. Bone Joint J.

[29] Sztramko R, Levinson AJ, Wurster AE, Jezrawi R, Sivapathasundaram B, Papaioannou A, Cowan D, St Onge J, Marr S, Patterson C, Woo T, Mosca L, Lokker C (2021). Online educational tools for caregivers of people with dementia: a scoping literature review. Can Geriatr J.

[30] Petrovic M, Gaggioli A (2020). Digital mental health tools for caregivers of older adults-a scoping review. Front Public Health.

[31] Truskinovsky Y, Finlay JM, Kobayashi LC (2022). Caregiving in a pandemic: COVID-19 and the well-being of family caregivers 55+ in the United States. Med Care Res Rev.

[32] Hughes MC, Liu Y, Baumbach A (2021). Impact of COVID-19 on the health and well-being of informal caregivers of people with dementia: a rapid systematic review. Gerontol Geriatr Med.

[33] Cohen G, Russo MJ, Campos JA, Allegri RF (2020). Living with dementia: increased level of caregiver stress in times of COVID-19. Int Psychogeriatr.

[34] Penteado CT, Loureiro JC, Pais MV, Carvalho CL, Sant'Ana LF, Valiengo LC, Stella F, Forlenza OV (2020). Mental health status of psychogeriatric patients during the 2019 new coronavirus disease (COVID-19) pandemic and effects on caregiver burden. Front Psychiatry.

[35] Newman K, Wang AH, Wang AZ, Hanna D (2019). The role of internet-based digital tools in reducing social isolation and addressing support needs among informal caregivers: a scoping review. BMC Public Health.

[36] Fabius CD, Wolff JL, Willink A, Skehan ME, Mulcahy J, Kasper J (2021). Community-based long-term services and supports: are the needs of older adults and their caregivers being met?. The Commonwealth Fund.

[37] Gaugler Joseph E, Kane Robert L, Kane Rosalie A, Newcomer Robert (2005). Early community-based service utilization and its effects on institutionalization in dementia caregiving. Gerontologist.

[38] Zhang Q, Lu J, Jin Y (2020). Artificial intelligence in recommender systems. Complex Intell Syst.

[39] National Alliance for Cargiving, American Association of Retired Persons (2020). Caregiving in the United States 2020. American Association of Retired Persons.

