# Supplementary material for: The Olera.care Digital Caregiving Assistance Platform for Dementia Caregivers: Preliminary Evaluation Study
Source: JMIR Aging. 2024 Apr 17;7:e55132. doi: 10.2196/55132 (PMC11063878; doi:10.2196/55132)
Supplement: Multimedia Appendix 1 [file aging_v7i1e55132_app1.docx]

**Table.** Two-sample T-Test Results for Platform Feature Evaluation by Participants Characteristics.

**
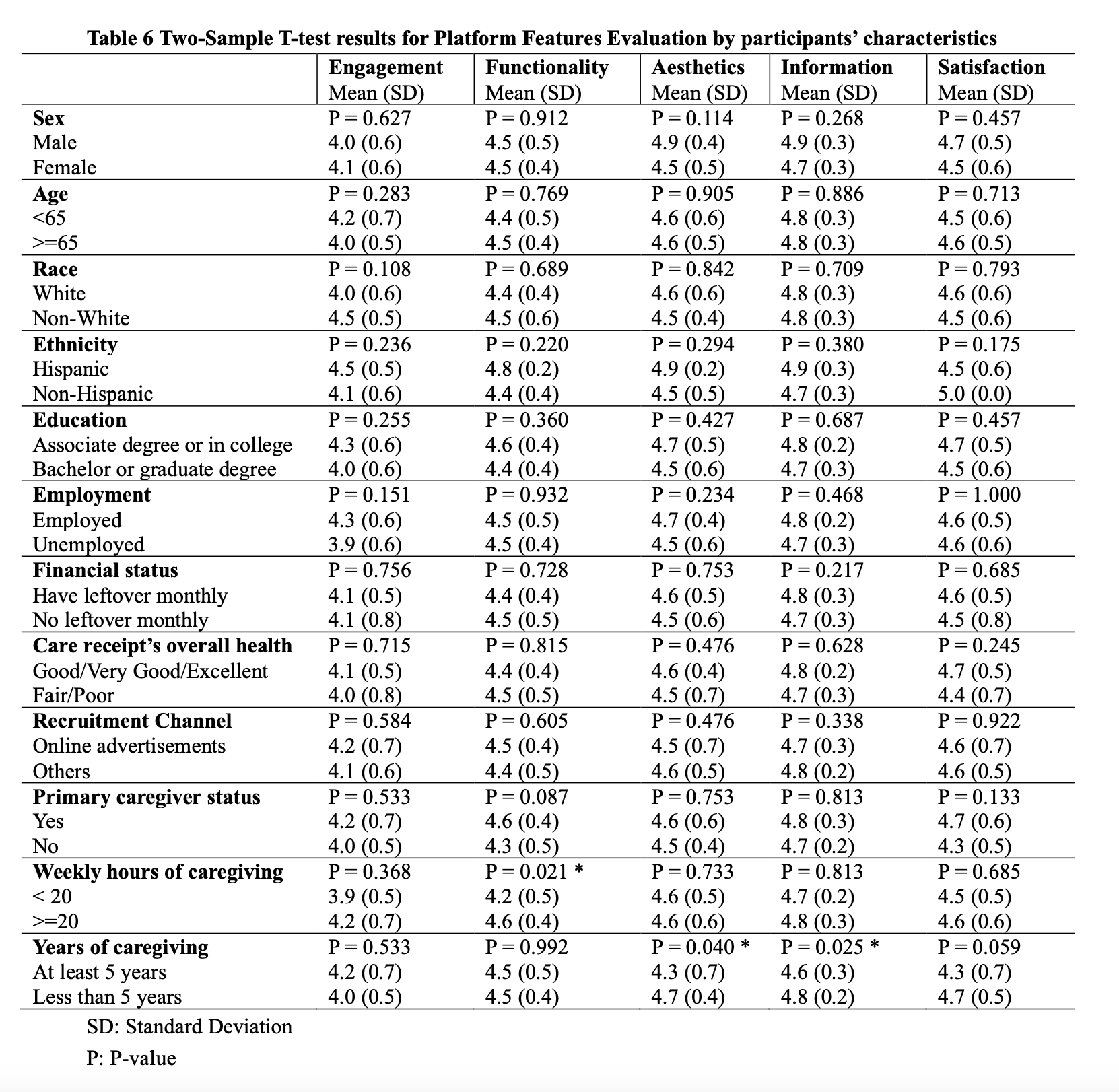
**
